# Source tracking of human leptospirosis: serotyping and genotyping of *Leptospira* isolated from rodents in the epidemic area of Guizhou province, China

**DOI:** 10.1186/1471-2180-13-75

**Published:** 2013-04-01

**Authors:** Shijun Li, Dingming Wang, Cuicai Zhang, Xiaoyu Wei, Kecheng Tian, Xiuwen Li, Yixin Nie, Ying Liu, Guanghai Yao, Jingzhu Zhou, Guangpeng Tang, Xiugao Jiang, Jie Yan

**Affiliations:** 1 Institute of Communicable Disease Control and Prevention, Guizhou Provincial Centre for Disease Control and Prevention, 73 Bageyan Road, Guiyang 550004, Guizhou, People's Republic of China; 2 National Institute for Communicable Disease Control and Prevention, Chinese Centre for Disease Control and Prevention, 155 Changbai Road, Changping District, 102206 Beijing, People's Republic of China; 3 Department of Medical Microbiology and Parasitology, College of Medicine, Zhejiang University, Hangzhou, People's Republic of China; 4 Laboratory of Microbiology and Parasitology, College of Medicine, Zhejiang University, 388 Yuhangtang Road, Hangzhou 310058, Zhejiang, People's Republic of China

**Keywords:** Rodents, *Leptospira*, Serotyping, Genotyping, Source of infection

## Abstract

**Background:**

Sustained human leptospirosis as well as death cases has been reported in Qiandongnan Prefecture, Southeast of Guizhou, China, recently, but these human patients were only clinically diagnosed, and leptospires have never been isolated from patients in these epidemic regions, In order to track the source of infection and understand the etiologic characteristic of leptospirosis, we performed rodent carrier surveillance for leptospirosis in the epidemic area in 2011. The population distribution of rodents in the epidemic regions was revealed.

**Results:**

Four strains of leptospire were isolated from *Apodemus agrarius*. Microscopic agglutination test (MAT) confirmed the four isolates belonged to leptospiral serogroup Icterohaemorrhagiae. Multilocus sequence typing (MLST) indicated that all the four strains were defined as sequence type 1(ST1), which is identical to the three strains isolated from *Rattus tanezumi* in Rongjiang County in 2007. Clustering analysis of the MLST data indicated that the local isolates exactly matched with reference strain of leptospiral serovar Lai strain 56601, which is consistent with anti-*Leptospira* antibody detection of patients using MAT.

**Conclusions:**

*Apodemus agrarius* may be the potentially important carrier of leptospirosis and the potential source of leptospiral infection in human, and serovar Lai maybe the epidemic serovar of *Leptospira* in the localities.

## Background

Leptospirosis is one of the most widespread zoonoses and is caused by infection with pathogenic spirochetes of the *Leptospira* genus [1]. Its incidence in humans is most frequent in developing countries, and the spectrum of human disease ranges from subclinical infection to severe symptoms of multiorgan disfunction with high case fatality rates, reaching mortalities as high as 70% in the case of severe pulmonary haemorrhage syndrome [2,3]. There is, for certain, an underestimation of the leptospirosis problem due to a lack of awareness and under-recognition through a lack of proper use of diagnostic tools [4].

*Leptospira* are maintained in the genital tract and renal tubules of wild and domestic animals and are excreted with urine into the environment where they can survive for several months depending on favorable conditions such as warm, humid environment with a neutral to slightly alkaline pH [5,6]. Rodents are recognized as important mammal reservoirs of *Leptospira* spp [7,8], which only present mild chronic disease or are asymptomatic, and shed infectious organisms in the urine for their lifetime [9]. Humans may be infected indirectly from animals by contact with contaminated water, soil or mud in a moist environment, or by direct contact with urine, fresh carcasses or organs [10]. Therefore, surveillance on carrier status of reservoir hosts and analysis on the characteristic of causative agents contribute to the clinic laboratory diagnosis, active surveillance, outbreak investigation and source tracking for leptospirosis.

Sustained human leptospirosis as well as death cases has been reported in Qiandongnan Prefecture, such as Jinping, Liping, and Rongjiang County, Southeast of Guizhou, in recent years [11]. According to the China National System for Disease Control and Prevention, twelve human leptospirosis cases with one death case were reported in Guizhou in 2011. However, *Leptospira* were never isolated from patients in recent years and the patients were only serologically diagnosed, and the etiologic characteristics of epidemic *Leptospira* remain unclear.

Traditionally, pathogenic *Leptospira* are classified into more than 200 serovars based on serological methods [12]. Nowadays, multilocus sequence typing (MLST) method has been recently proved for typing leptospires [4,13-15]. MLST is a simple PCR based technique, which makes use of automated DNA sequencers to assign and characterize the alleles present in different target genes. The selected loci are generally the housekeeping genes, which evolve very slowly over an evolutionary time-scale [4,16] and hence qualify as highly robust markers of ancient and modern ancestry. The sequencing of multiple loci provides a balance between technical feasibility and resolution.

In order to track the source of infection and understand the etiologic characteristics of human leptospirosis in the epidemic area, we performed rodent carrier status surveillance in Jinping, Liping and Rongjiang County in 2011. Leptospiral isolates were serologically and molecularly identified and typed using MAT and MLST, respectively. Our results will contribute to the prevention and control of leptospirosis in the localities.

## Methods

### Rodents collection

The present study was conducted in three sites including Jinping, Liping and Rongjiang County, where a high number of leptospirosis cases and deaths were reported in recent years.

Rodents were trapped by using Trap-night method [17] (traps with peanut bait) in rice-field environments in Jinping and Liping county, during September to October. Traps were placed at evening and fetched back at the next morning. Trapped rodents were identified by genus, species, and gender based on phenotypic characteristics (ears, body, tail, fur colour and sex) [17]. Rodents were dissected to collect kidneys. Live animals were killed by decapitation under anesthesia by diethyl ether. Kidney tissue samples were collected for isolation and culture of leptospires. Animal protocols were approved by the Animal Ethics Review Committee of Guizhou Provincial Centre for Disease Control and Prevention.

### Leptospiral isolation and cultivation

Freshly isolated kidney sample were inoculated to 8 mL liquid Ellinghausen - McCullough - Johnson - Harris (EMJH) medium (Difco, USA) [18]. Cultures were incubated at 28°C and evaluated weekly by dark field microscopy for up to 2 months [19]. *Leptospira* isolates and reference strains belonging to the Chinese 15 serogroups 15 serovars provided by Chinese Centre for Disease Control and Prevention (Chinese CDC) were cultivated at 28°C in Ellinghausen-McCullough-Johns on-Harris (EMJH) (Difco Laboratories, Detroit, MI, USA) liquid medium supplemented with 8% heat-inactivated rabbit serum [17].

### MAT

For the serogroup identification of leptospiral isolates, Microscopic agglutination test (MAT) was performed using a battery of anti-serum against the Chinese reference strains belonging to 15 serovars in 15 serogroups provided by Chinese CDC [20]. For detecting anti-*Leptospira* antibodies of serum samples (LCB, LH, ZJD, YCX, LJP, YZM, WSZ, LJX, and LDL) collected from patients in the local regions, MAT was carried using a battery of pathogenic reference strains belonging to Chinese 15 serovars in 15 serogroups of pathogenic *Leptospira* including leptospiral strains isolated in the epidemic area. The MAT titre was expressed as the reciprocal of the highest serum dilution that resulted in 50% agglutination of leptospires. The samples with titres ≥100 were recognized as positive.

### MLST analysis

DNA was extracted from cultures of *Leptospira* strains using DNA Extraction Kit (SBS Genetech, Beijing, China) according to the manufacturer’s directions. Seven loci (pntA, sucA, fadD, tpiA, pfkB, mreA, and glmU) were selected based on performance of primers as previously described (also can be obtained from the sharing website: http://leptospira.mlst.net) [21]. Primer sequences are shown in Table 1. Amplifications were performed in 50 μl total volumes of PCR reaction system contained approximately 25 μl of PreMix Taq (TaKaRa, Otsu, Japan), 2 μl of forward and reverse primers with concentrations of 10 pmol/μl, 2 μl of DNA, 19 μl of deionized water, respectively. Amplification was performed on an Biometra TProfession thermocycler (Biometra, Goettingen, Germany) using amplification parameters included an initial denaturation at 94°C for 5 minutes, followed by 30 cycles of 94°C for 10 seconds, 52°C (mreA, pfkB, pntA, sucA, and tpiA), or 50°C (fadD and glmU) for 15 seconds, 72°C for 50 seconds, then 72°C for 7 minutes. Following the standard MLST protocol, the PCR products were detected by electrophoresis of 1μl of each reaction on a 1.2% agarose gel for 30 min at 100 V, and were sequenced by ABI PRISM 377 DNA sequencer. Each allele was assigned a different allele number and the allelic profile (string of seven integers) was used to define the sequence type (ST). A *Leptospira* mlst website was established to provide public access to these data, and to provide a resource to other investigators who can use this to assign the ST of further strains. This can be accessed at http://leptospira.mlst.net.

**Table 1:** Information of loci proposed for MLST of leptospiral isolates

**Gene**	**Size of PCR product (bp)**	**Primer 5’-3’**	**Annealing temperature (°C)**
pntA	638	F: TGCCGATCCTACAACATTA	52
		R: AAGAAGCAAGATCCACAACTAC	
sucA	560	F: AGAAGAGGCCGGTTATCATCAG	52
		R: CTTCCGGGTCGTCTCCATTTA	
pfkB	560	F: CCGAAGATAAGGGGCATACC	52
		R: CAAGCTAAAACCGTGAGTGATT	
tpiA	534	F: AAGCCGTTTTCCTAGCACATTC	52
		R: AGGCGCCTACAAAAAGACCAGA	
mreA	602	F: AAAGCGGCCAACCTAACACC	52
		R: CGATCCCAGACGCAAGTAAG	
glmU	557	F: GGAAGGGCACCCGTATGAA	50
		R: TCCCTGAGCGTTTTGATTT	
fadD	577	F: AGTATGGCGTATCTTCCTCCTT	50
		R: TTCCCACTGTAATTTCTCCTAA	

## Results

### Rodent distribution

A total of 160 rodents including *Apodemus agrarius*, *Rattus norvegicus*, *Apodemus chevrieri*, *Rattus rattus sladerni*, *Rattus nitidus*, *Hodgson*, *Rattus flavipectus*, and other rodents were trapped, and the prevalent rodent for Jinping and Liping was *Apodemus agrarius*, with 37.8% of the total rodents for Jinping and 21.9% for Liping, while no *Apodemus agrarius* was trapped in Rongjiang County, in which *Apodemus chevieri* was the prevalent rodents (54.8%) (Table 2).

**Table 2:** Rodent distribution and leptospiral carrier status in the epidemic area of Guizhou Province

**Distribution of rodents and statistics of rodent surveillance**		**Data of rodents for the three sites**		
		**Jinping**	**Liping**	**Rongjiang**
Distribution of rodents	*Apodemus agrarius*	17*	16#	0
	*Rattus norvegicus*	2	2	0
	*Apodemus chevrieri*	3	40	20
	*Rattus tanezumi*	13	3	0
	*Rattus nitidus Hodgson*	3	0	0
	*Rattus flavipectus*	1	4	11
	Other rodents	6	8	11
Statistics of rodent monitoring	Number of traps (NT)	900	600	600
	Number of trapped rodents (NR)	45	73	42
	Percentage of rodents density (NR/NT)	5	12.7	7
	Number of isolated strains (NS)	3	1	0
	Percentage of positive isolation (NS/NR)	6.7	1.4	0

* Three strains of leptospire were isolated from seventeen *Apodemus agrarius*.

^#^ One strain of leptospire was isolated from sixteen *Apodemus agrarius*.

### Carrier status of rodents

Three strains of spirochetes (nominated as JP13, JP15 and JP19) were isolated from *Apodemus agrarius* in Jinping County, with positive rates of 6.7% (3 strains isolated from 45 rodents), and one strain (nominated as LP62) from *Apodemus agrarius* in Liping County, with positive rates of 1.4% (1 strain isolated from 73 rodents). No spirochetes were isolated from the sites in Rongiang County. All the other species of rodents were negative for spirochete isolation, except for *Apodemus agrarius* in the three sites. The positive isolation rates of spirochete from *Apodemus agrarius* was 17.65% (3 strains isolated from 17 *Apodemus agrarius*) for the site in Jingping, and 6.25% (1 strain isolated from 16 *Apodemus agrarius*) for the site in Liping (Table 2).

### Results of serogroup identification of leptospiral isolates

MAT was performed using a battery of anti-serum against the Chinese reference strains belonging to 15 serovars in 15 serogroups. All the four strains agglutinated with anti-serum against reference strain 56601 belonging to serovars Lai of serogroup Icterohaemorrhagiae with titres ≥100, and no positive results of MAT were observed with anti- serum against to strains belong to the other serogroups (Table 3), according to the determine standard that samples with titres ≥100 were recognized as positive.

**Table 3:** **Results of MAT identification for leptospires isolated from *Apodemus agrarius *in Guizhou Province**

**Anti-serum against the Chinese reference strains belonging to 15 serovars in 15 serogroups**				**MAT results (titres) of isolated strains**			
**Anti-Serum No.**	**Strain**	**Serovar**	**Serogroup**	**JP13**	**JP15**	**JP19**	**LP62**
56601	Lai	Lai	Icterohaemorrhagiae	+ (1:800)	+ (1:800)	+ (1:800)	+ (1:400)
56602	M10	Javanica	Javanica	-	-	-	-
56603	Lin	Canicola	Canicola	-	-	-	-
56604	Pishu	Ballum	Ballum	-	-	-	-
56605	4	Pyrogenes	Pyrogenes	-	-	-	-
56606	Lin 4	Autumnalis	Autumnalis	-	-	-	-
56607	Sep-65	Australis	Australis	-	-	-	-
56608	Luo	Pomona	Pomona	-	-	-	-
56609	Lin 6	Linhai	Grippotyphosa	-	-	-	-
56610	P7	Hebdomadis	Hebdomadis	-	-	-	-
56612	L37	Paidjian	Bataviae	-	-	-	-
56613	65-52	Tarassovi	Tararrovi	-	-	-	-
56615	L 105	Cingshui	Manhao	-	-	-	-
56635	L 138	Sejroe	Wolffi	-	-	-	-
56655	Nan 10	Mini	Mini	-	-	-	-

+: Positive; -: Negative.

### MLST pattern of leptospiral isolates

**Figure 1:**
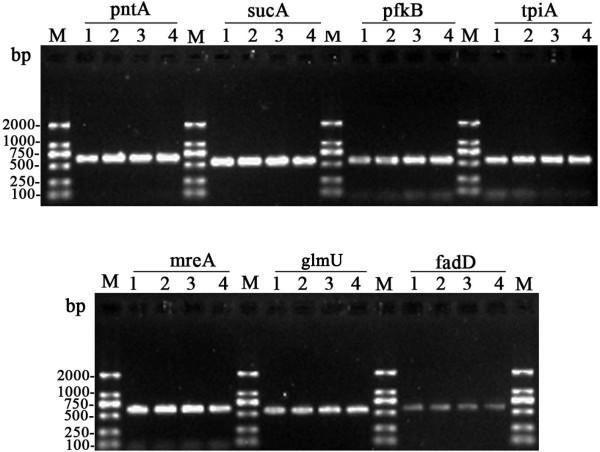
**PCR products from the seven selected MLST loci of four leptospiral strains isolated from Jinping and Liping County, Guizhou province.** PCR products were electrophoresised through a 1.2% agarose gel. M: 100 bp DNA Ladder; 1, *Leptospira* isolate JP13; 2, *Leptospira* isolate JP15; 3, *Leptospira* isolate JP19; 4, *Leptospira* isolate JP62.

Seven MLST loci based primers were used to amplify the chromosome DNA of leptospiral isolates, and all of the seven loci were successfully amplified from the four isolates. The MLST pattern showed that the four isolates produced a same size of PCR segment at the same locus (Figure 1).

### ST of leptospiral isolates

**Figure 2:**
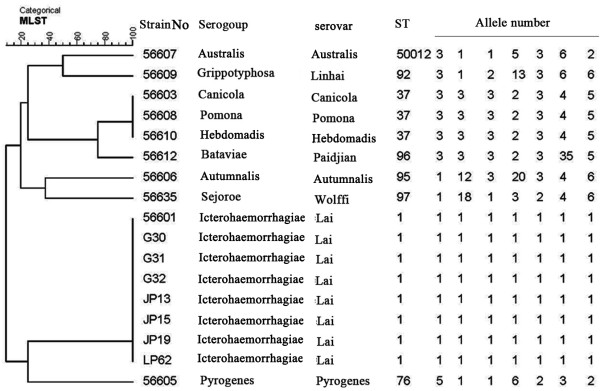
**Dendrogram of MLST data showing *Leptospira *isolates and *L. interrogans *strain Lai clustering together at 100%.**

Seven loci (pntA, sucA, fadD, tpiA, pfkB, mreA, and glmU) of the chromosome DNA of the four leptospiral isolates were successfully sequenced. The sequences were analysed following the standard MLST protocol which can be accessed at http:// leptospira.mlst.net, an allele number was assigned to all the allele of different leptospiral strains and the allelic profile (string of seven integers) was defined as sequence type 1 (ST1: 1-1-1-1-1-1-1) (Figure 2). According to the ST profile, all of the three leptospiral strains exactly matched with *Leptospira* serogroup Icterohaemorrhagiae Serovar Lai strain 56601.

### Clustering analysis results

Clustering analysis (Figure 2), based on the MLST data of the four leptospiral strains isolated from *Apodemus agrarius* in 2011, three strains isolated in 2007 and ten reference strains representative to the ten common epidemic serogroups provided by Chinese CDC, suggested that all the 7 leptospiral isolates and reference strain 56601 formed one clade, which indicated that all the leptospiral isolates including four strains isolated from *Apodemus agrarius* in 2011 and three strains isolated from *Rattus tanezumi* in 2007 are typed as *Leptospira* serogroup Icterohaemorrhagiae Serovar Lai.

### Anti-*Leptospira* antibody of suspected patients

MAT were performed using serum samples of suspected patients with Chinese reference strains belonging to 15 serovars in 15 serogroups of pathogenic *Leptospira* species and the four leptospiral isolates. Of the 9 serum samples of patients tested by MAT, 66.7% (6/9) had agglutinating antibodies against isolate JP13, JP15, JP19 and LP62 and reference strain 56601, but not reference strains belong to other serogroups (Table 4).

**Table 4:** **Results of MAT detection for anti-*Leptospira *antibody of patients from epidemic region**

**Isolates and reference strains**				**Serum samples of patients and Agglutination results (titres)**								
**NO.**	**Strain**	**Serovar**	**Serogroup**	**LCB**	**LH**	**ZJD**	**YCX**	**LJP**	**YZM**	**WSZ**	**LJX**	**LDL**
JP13	JP13	/*	Icterohaemorrhagiae	+ (1:800)	+ (1:800)	-	-	+ (1:400)	+ (1:800)	+ (1:800)	+ (1:800)	-
JP15	JP15	/*	Icterohaemorrhagiae	+ (1:800)	+ (1:800)	-	-	+ (1:400)	+ (1:800)	+ (1:800)	+ (1:800)	-
JP19	JP19	/*	Icterohaemorrhagiae	+ (1:800)	+ (1:800)	-	-	+ (1:400)	+ (1:800)	+ (1:800)	+ (1:400)	-
LP62	LP62	/*	Icterohaemorrhagiae	+ (1:400)	+ (1:400)	-	-	+ (1:400)	+ (1:400)	+ (1:400)	+ (1:400)	-
G30	G30	/*	Icterohaemorrhagiae	+ (1:400)	+ (1:400)	-	-	+ (1:200)	+ (1:800)	+ (1:400)	+ (1:400)	-
G31	G30	/*	Icterohaemorrhagiae	+ (1:400)	+ (1:400)	-	-	+ (1:200)	+ (1:800)	+ (1:400)	+ (1:400)	-
G32	G30	/*	Icterohaemorrhagiae	+ (1:400)	+ (1:400)	-	-	+ (1:200)	+ (1:800)	+ (1:400)	+ (1:400)	-
56601	Lai	Lai	Icterohaemorrhagiae	+ (1:800)	+ (1:800)	-	-	+ (1:400)	+ (1:800)	+ (1:800)	+ (1:400)	-
56602	M10	Javanica	Javanica	-	-	-	-	-	-	-	-	-
56603	Lin	Canicola	Canicola	-	-	-	-	-	-	-	-	-
56604	Pishu	Ballum	Ballum	-	-	-	-	-	-	-	-	-
56605	4	Pyrogenes	Pyrogenes	-	-	-	-	-	-	-	-	-
56606	Lin 4	Autumnalis	Autumnalis	-	-	-	-	-	-	-	-	-
56607	Sep-65	Australis	Australis	-	-	-	-	-	-	-	-	-
56608	Luo	Pomona	Pomona	-	-	-	-	-	-	-	-	-
56609	Lin 6	Linhai	Grippotyphosa	-	-	-	-	-	-	-	-	-
56610	P7	Hebdomadis	Hebdomadis	-	-	-	-	-	-	-	-	-
56612	L37	Paidjian	Bataviae	-	-	-	-	-	-	-	-	-
56613	65-52	Tarassovi	Tararrovi	-	-	-	-	-	-	-	-	-
56615	L 105	Cingshui	Manhao	-	-	-	-	-	-	-	-	-
56635	L 138	Sejroe	Wolffi	-	-	-	-	-	-	-	-	-
56655	Nan 10	Mini	Mini	-	-	-	-	-	-	-	-	-

* MAT cannot identify the isolates to Serovar level; +: Positive; +: Negative.

## Discussion

The present study performed surveillance on rodent carrier status of *Leptospira* in the epidemic area in 2011. The population distribution of rodents in the epidemic regions was revealed and four strains of leptospire were isolated from *Apodemus agrarius*. MAT confirmed the four isolates belonged to leptospiral serogroup Icterohaemorrhagiae. MLST define the four isolated as ST1 and exactly matched with reference strain of leptospiral serovar Lai strain 56601, which is consistent with anti-*Leptospira* antibody detection of patients using MAT. Together, these findings indicate that *Apodemus agrarius* may be the potentially important carrier of leptospirosis for Jinping and Liping County, and serovar Lai maybe the epidemic serovar of *Leptospira* in the epidemic area. Our results will contribute to the control and prevention of leptospirosis in the localities.

Guizhou has been proved the old foci of leptospirosis in China [11,22,23]. Qiandongnan Prefecture of Guizhou province was the high-incidence area of leptospirosis in Guizhou Province. For example, 14 126 human leptospirosis cases with 534 deaths were reported in Qiannan prefecture from 1958 to 2005. Investigation on the epidemiology of Leptospirosis in Liping county revealed that a total of 127 leptospirosis cases with 28 deaths were reported from 2001 to 2008 [11]. According to the China National System for Disease Control and Prevention, there were several cases of leptospirosis patients as well as death cases were reported in Guizhou Province in every year of recent years. For instance, twelve human leptospirosis cases with one death case were reported in Guizhou in 2011. However, the leptospires were never isolated from human and animal in recent years, the reason for the failure of pathogen isolation maybe the using of antibiotics for treatment before collecting samples such as urine and blood from patients, or there is, for certain, an underestimation of the leptospirosis problem due to lack of awareness or experiences, so, these reported cases were only clinically diagnosed, and the source of infection and the characteristic of pathogen remain unclear.

In order to track the source of human leptospirosis, we chose three sites located in Jingping, Liping and Rongjiang County, respectively, the high incidence county of human leptospirosis, to perform surveillance on carrier status of *Leptospira* in rodents which has been proved as the important mammal reservoirs of *Leptospira* spp. [7,8]. Four leptospires were isolated from *Apodemus agrarius*, which is consistent with previous study that the *Apodemus agrarius* was a very important reservoir host of leptospirosis in Guizhou province. To understand the etiologic characteristic of the isolates, both the classic MAT methods and the molecular method (MLST) which is demonstrated suitable for the typing of common epidemic leptospires in China [21] were applied to identify the four isolates. MAT indicated that the four isolates belonged to leptaspiral serogroup Icterohaemorrhagiae, while MLST revealed the four isolates exactly matched with Serovar Lai strain 56601 belonging to serogroup Icterohaemorrhagiae and the result of MAT was consistent with that of MLST.

To establish a linkage of the isolates with the patients in the epidemic area as well as to give a laboratory evidence for the diagnosis of leptospirosis in the patients, serum samples were collected from patients in the epidemic area for the detection of anti-*Leptospira* antibody using MAT, the results showed that 66.7% (6/9) of the serum samples of patients had agglutinating antibodies against isolate JP13, JP15, JP19 and LP62 isolates and reference strain 56601, but not reference strains belong to other serogroups, which is consistent with the typing results of leptospiral isolates. It implies that *Apodemus agrarius* may be the main carrier of *Leptospira* in Jinping and Liping County, serovar Lai maybe the cause for the human leptospirosis in the epidemic area in Guizhou province.

## Conclusion

Rodent carrier surveillance for leptospirosis was performed in the epidemic area of Guizhou in 2011. The results showed that *Apodemus agrarius* may be the potentially important carrier of leptospirosis and the potential source of leptospiral infection in human, and serovar Lai maybe the epidemic serovar of *Leptospira* in the localities.

## Competing interests

The authors declare that they have no competing interests.

## Authors’ contributions

SL executed the Leptspiral isolation, MAT, PCR and MLST experiments, analyzed the data and drafted the manuscript; CZ participated in the analysis of MLST results; DW participated in the study design; XW participated the MLST experiments; KT participated in the rodents Trapping; XL and XJ provided the reference strains of *L. interrogans;* YN provided the rabbit anti-*Leptospira* serum; YL contributed to the culture of leptospiral strains and the MAT experiments; GY and JZ participated in rodents trapping and *Leptospira* isolation. GT participated in the study design; JY critically revised the manuscript; all authors read and approved the final manuscript.
